# The past, present and future use of epidemiological intelligence to plan malaria vector control and parasite prevention in Uganda

**DOI:** 10.1186/s12936-015-0677-4

**Published:** 2015-04-15

**Authors:** Ambrose O Talisuna, Abdisalan M Noor, Albert P Okui, Robert W Snow

**Affiliations:** INFORM Project, Department of Public Health Research, KEMRI-Welcome Trust Research Programme, Nairobi, Kenya; Centre for Tropical Medicine & Global Health, Nuffield Department of Clinical Medicine, University of Oxford, CCVTM, Oxford, UK; Uganda Malaria Surveillance Programme (UMSP), Kampala, Uganda; Ministry of Health, National Malaria Control Programme, Plot 6 Lourdel Road Nakasero, Kampala, Uganda

**Keywords:** Epidemiological intelligence, Surveillance, Monitoring and evaluation, Vector control, Parasite prevention, Malaria control, Global malaria eradication project, Malaria elimination, Roll Back Malaria, Uganda

## Abstract

**Background:**

An important prelude to developing strategies to control infectious diseases is a detailed epidemiological evidence platform to target cost-effective interventions and define resource needs.

**Methods:**

A review of published and un-published reports of malaria vector control and parasite prevention in Uganda was conducted for the period 1900–2013. The objective was to provide a perspective as to how epidemiological intelligence was used to design malaria control before and during the global malaria eradication programme (GMEP) and to contrast this with the evidence generated in support of the Roll Back Malaria (RBM) initiative from 1998 to date.

**Results:**

During the GMEP era, comprehensive investigations were undertaken on the effectiveness of vector and parasite control such as indoor residual house-spraying (IRS) and mass drug administration (MDA) at different sites in Uganda. Nationwide malariometric surveys were undertaken between 1964 and 1967 to provide a profile of risk, epidemiology and seasonality leading to an evidence-based national cartography of risk to characterize the diversity of malaria transmission in Uganda. At the launch of the RBM initiative in the late 1990s, an equivalent level of evidence was lacking. There was no contemporary national evidence-base for the likely impact of insecticide-treated nets (ITN), no new malariometric data, no new national cartography of malaria risk or any evidence of tailored intervention delivery based on variations in the ecology of malaria risk in Uganda.

**Discussion:**

Despite millions of dollars of overseas development assistance over the last ten years in ITN, and more recently the resurrection of the use of IRS, the epidemiological impact of vector control remains uncertain due to an absence of nationwide basic parasite and vector-based field studies.

**Conclusion:**

Readily available epidemiological data should become the future business model to maximize malaria funding from 2015. Over the next five to ten years, accountability, impact analysis, financial business cases supported by a culture of data use should become the new paradigm by which malaria programmes, governments and their development partners operate.

## Background

Over the last decade Uganda has received over US$600 million in overseas development assistance from multilateral and bilateral development partners to control malaria. The interest in malaria as an impediment to Uganda’s development was first highlighted during the colonial era [1,2]. Later, in 1950, the first malaria conference in Equatorial Africa, convened by the World Health Organization (WHO), was held in Kampala, Uganda [3-5]. This landmark conference was used to present and assess all the available information on the epidemiological aspects of malaria and attempted to coordinate the various methods of research and control of the disease. One major recommendation of the conference was that the benefits that malaria control might bring to the indigenous populations should be evaluated [3-5]. The role of research as a means to address the technical problems of malaria control in tropical Africa was integral to the Global Malaria Eradication Programme (GMEP) effort. The 1950 Kampala conference catalyzed many studies and epidemiological investigations in Africa, including a number of important field trials aimed at the ‘eradication’ (appropriate term should have been elimination) of malaria.

However, after 1969, efforts to control the disease were disrupted when the WHO put on hold the goal of malaria eradication [6]. From the early 1970s to the late 1980s, political and economic turmoil curtailed any reasonable malaria control in Uganda. Renewed interests in malaria control in the country were started in the 1990s, after the endorsement of the global malaria strategy in 1993 [7] and were initially supported by the World Bank [8]. Coincidentally, during this period, treatments for malaria started failing and the country found itself with repeated malaria epidemics and an escalating disease burden [9,10]. In 1998, the Roll Back Malaria (RBM) initiative was launched [11], followed, in 2002, by opportunities to access new funding through the Global Fund [12].

### Methods and literature abstraction

Published and un-published reports about malaria vector control and parasite prevention in Uganda for the period 1900 to 2013 were reviewed. The objectives were to provide a historical overview and evolution of malaria vector control and parasite prevention from the pre-eradication period to the era of the Global Malaria Eradication Programme (GMEP) to the present Roll Back Malaria-RBM control period. This review was motivated by a need to: a) capture a historical perspective of malaria vector control and parasite prevention to draw lessons for today’s control/elimination ambitions; and b) maintain an institutional memory of the past efforts of malaria vector control and parasite prevention in Uganda - who was involved, what was done, what worked and more importantly what did not work.

Online electronic literature databases were used as one means for identifying peer-reviewed, published papers. Due to its wide coverage of the biomedical literature, PubMed was used as the basis for all the initial online searches of published sources. In addition, the World Health Organization Library Database and the African Journals Online (AJOL) was used to search for relevant literature. In all digital electronic database searches for published work the free text keywords “*malaria*” and “*Uganda*” were used. Specialised Medical Subject Headings (MeSH) terms in digital archive searches were avoided to ensure as wide as possible search inclusion. Further, national malaria control programme documents at the ministry of health headquarters in Kampala, Uganda were reviewed. Finally, in 2013, in partnership with the national malaria control programme (NMCP); all available epidemiological data on malaria infection prevalence were assembled to map malaria risk in Uganda at a resolution of 112 districts to support the future analysis of change since 2000. The statistical models used to derive the Uganda malaria risk map are already published [13]. The review further describes how research evidence and epidemiological intelligence was used to shape policy and design malaria control in Uganda during the GMEP period and compares and contrasts this with the evidence generated over 50 years later in support of the RBM initiative.

## Review findings

### Defining the malaria burden in Uganda 1929–1950

The precise burden of malaria and its distribution countrywide was unclear to those responsible for health statistics between the two World Wars [1]. As stated in the 1927 Annual Report of the Medical Department “*The very low number of deaths recorded under clinical malaria, i.e. malaria in which the diagnosis was not confirmed by the microscope, is explained by the fact that most of these cases were mild and attended to as out-patients, when the facilities for microscopic diagnosis were not always possible*” [2].

After the Second World War, malaria epidemics were noted in the township areas, despite being ‘protected’ by permanent drainage schemes and routine oiling [2]. At Kigezi (presently composed of four small districts: Kabale, Rukungiri, Kanungu, and Kisoro), a highland area in south-western Uganda, reports emerged of malaria cases from areas previously considered free of the disease [2]. However, by 1950, a review of statistics suggested that malaria mortality among hospital admissions might have declined over the previous 20 years among Europeans and Asians, and it was an unusual event to become infected with malaria in some of the major towns. Although European settled areas had come under some degree of control, the 1950 Annual Report noted as follows “*the available evidence suggests that no appreciable change has taken place in its incidence among general African population*” [2]. The precise malaria burden among the entire Ugandan population continued to remain an elusive statistic even up to 1950, not least because 80% of the diagnoses of malaria at hospitals were unsupported by microscopic examination [2].

### Malaria eradication experimental pilot projects

It was not until the late 1950s, after the WHO Kampala Conference, that a concerted effort was mounted to understand the complex epidemiology of malaria in relation to its control. ‘Eradication’ experimental pilot projects were supported by rigorous evaluation with pre- *versus* post-intervention trials of vector and parasite-based control at four locations (Kigezi in south western, Masaka in Central, Lugazi and Kakira in Eastern Uganda).

#### Northern Kigezi (1959–1964)

Between 1957 and 1958, comprehensive human and vector-based studies were undertaken to characterize the epidemiology of malaria in a government resettlement scheme of approximately 50,000 people in northern Kigezi district [14]. In 1959, the experimental intervention studies focussed on three annual rounds of indoor residual spraying (IRS) with dichloro-diphenyl-trichloroethane (DDT), 2 g per sq m of sprayed surface, and three rounds of mass drug administration (MDA) with single doses of chloroquine (200 mg base) and pyrimethamine (16.5 mg base) (CQ/P). By 1959, the project had been extended to the entire Kigezi district [14]. An important component of these interventions was monitoring and evaluation (M & E), including: community and school surveys of infection prevalence by species and spleen rates among children aged two to nine years; monthly infant parasite prevalence surveys at dispensaries and health posts before and after spraying; monthly fever surveys during visits to the health facilities; sampling adult indoor-resting mosquitoes, outdoor-resting traps for outdoor catches; larval searches as part of reconnaissance surveys; use of experimental huts; establishment of laboratory colonies; and, breeding of mosquitoes under uniform conditions for bioassay tests and experimental work. In addition, a series of bioassay tests were carried out in the experimental huts to ascertain the decline of the toxic effect of DDT deposits, as well as susceptibility tests to determine the susceptibility of adult mosquitoes and larvae to insecticides [14]. Further, the field teams collected robust operational coverage data of every single human dwelling and animal shelter and the composition of spraying squads [14].

*Anopheles gambiae s.l.* was the main vector throughout the Kigezi area, with *Anopheles funestus* playing a secondary role in malaria transmission in a few localities [14,15]. During the first year of the operations, there was an almost complete disappearance of malaria: parasite prevalence among children aged two to nine years declined from 16.6 to 0.3%; the number of *An. gambiae* and *An. funestus* found resting in houses, which was, at baseline, between 26.5 and 43.5 per house, respectively, was reduced to practically nil after DDT spraying. However, the outdoor-resting vector populations remained unchanged during the first six months, but were substantially reduced thereafter and only *An. funestus* was occasionally found, suggesting a six-month lag between reductions of indoor- and outdoor-resting vector populations. The average number of monthly *An. gambiae* entering treated huts and caught in window traps declined by two-thirds after nine months. Surveys carried out in neighbouring districts showed practically no reduction in malaria rates [14,16]. By 1963, the malaria eradication experiment in North Kigezi was deemed a success, having eliminated malaria (reduction in parasite rate by a factor of 50 to 1) from almost the entire district [14]. However, imported malaria led to localized outbreaks in the later stages of the scheme because malaria was never completely interrupted in the areas adjoining the protected areas of North Kigezi [14].

#### Kigezi highlands (1960–1964)

Following the success of the campaign in North Kigezi, and an attempt to prevent the rate of new infections being brought into the protected areas, it was decided to extend the eradication operations to the whole of Kigezi district. The malaria ‘eradication’ measures used in the Lake Bunyonyi area were similar to those used in the North Kigezi programme [14,17] with the exception that there were two rounds instead of three of MDA with CQ/P. The results of the Lake Bunyonyi campaign were equally impressive: not a single *An. funestus* was observed in six surveys conducted after DDT spraying, from February 1961 to May 1962. In spite of the complete elimination of *An. funestus* from the area, other less dominant anophelines were implicated in transmission, including: *Anopheles coustani*, *Anopheles listeri, Anopheles marshalli,* and *Anopheles kingi* [17]. Parasite prevalence in children aged two to nine years declined from 21% in 1959 to 0.1% in 1960/1961. De Zulueta and colleagues concluded as follows: “*the results so far obtained in Kigezi indicate the possibility of malaria “eradication” in the area within a short time. Preliminary surveys in other parts of Western and Central Uganda indicate that no more malaria is to be found there than in Kigezi, thus showing that the “eradication” of malaria in Central and Western Uganda is feasible*” [17].

#### Field trial of Malathion (S-[1, 2-di-(ethoxycarbonyl) ethyl] O, O-dimethyl phosphorodithioate, in Masaka district (1963–1964)

Between 1963 and 1964, with the assistance of the WHO, a large-scale field trial of malathion was carried out in Masaka district [18]. The area of intervention covered 500 sq km and a population of 26,000 inhabitants. All houses and animal shelters were sprayed with malathion approximately every four months. Epidemiological evaluations were organized from November 1964 across a central area comprising 40% of the sprayed areas using parasitological and entomological methods developed during the Kigezi project. The average combined densities of *An. funestus* and *An. gambiae*, in the sprayed area, declined from 66 per shelter per day in the pre-trial period 1960–1961 to 0.0011 at the end of 1964. No significant changes were noted in the non-sprayed areas. However, the persistence of malaria cases and the observation of some infections in infants born after the commencement of spraying were reported as not consistent with the entomological observations. Further epidemiological investigations revealed that there were constant movements of the population in and out of the small intervention area and consequently a high rate of autochthonous cases due to imported infections [18].

#### Chloroquine-medicated salt for malaria suppression Lugazi and Kakira (1964–1965)

A mixture of CQ with common salt had been proposed as an alternative method for MDA in situations where insecticide spraying was not effective [19]. A trial began in Uganda between 1964 and 1965, involving the free distribution of CQ-medicated salt to workers and their dependants at two sugar estates, Lugazi and Kakira in Eastern Uganda [20]. Prior to the launch of the trial, it had been shown that IRS using DDT was unsuccessful in removing malaria infection in the highly mobile immigrant labour force at these estates that recruited people from all over Uganda and Rwanda and employed circa 10,000 people on each estate. Weekly administration of CQ tablets had been tried but abandoned due to widespread objections to the bitter taste. The effects of the MDA programme were assessed using mass blood examinations and morbidity data comparing recipient populations with similar populations on the estate not receiving the medicated salt. The results of this pilot experiment were inconclusive. There were no differences in infection or morbidity among children during 1964; larger differences were observed by 1965 notably among adults, a 30% decline in morbid events, but not among infants. There were problems with irregular distribution of CQ-medicated salt and some households bought alternative salt supplies from shops to avoid the bitter taste. Overall, it was concluded that with the satisfactory distribution of CQ-medicated salt, significant reductions in the crude parasite rates and morbidity could be achieved. Failure to achieve complete suppression because of operational shortcomings, individual non-cooperation and inadequate uptake of the medicated salt meant that the wider adoption of this strategy was never pursued [20].

#### Developing an epidemiological profile of malaria risk (1965–1967)

Building an epidemiological profile formed a major effort of the mid-1960s ‘pre-eradication’ effort, resulting in intensive and extensive investigations in some areas and wider mapping of risk across the country. The largest surveys were undertaken in Kigezi (presently composed of four small districts), Masaka district (presently composed of eight small districts), Busoga district currently composed of ten smaller districts, and Karamoja district currently composed of seven smaller districts [14-17,21-23]. For reasons not entirely clear, the central region was excluded. During these surveys, the field teams described the topography, the climate including temperature, humidity, winds, peak rainfall, and the main agricultural pre-occupation of surveyed localities. Field operations were initiated in February 1964 and continued until the end of 1967.

Malariometric, monthly infant and fever surveys and entomological surveys were carried out at regular intervals at ten selected localities in each district (except Karamoja where 15 sites were selected). School surveys were also carried out at all the sites at approximately three-monthly intervals, while general mass population surveys were conducted at eight of the ten localities at six-monthly intervals. Blood films were taken by medical assistants in charge of the medical units and collected every fortnight by project staff for examination at a central laboratory at the district headquarters. Eight indicator localities were selected in each district for monthly entomological surveys; including: daytime spray catches in eight fixed houses of different types of structures; all human and animal landing night catches indoors and outdoors for two consecutive nights (four localities); window trap observations (four localities); identification of the blood digestion stages and salivary gland dissections; identification of blood meals from samples collected in different types of structures [21-24]. These were some of the most significant national examinations of the epidemiology of malaria risk in Africa at the time.

The national patterns of malaria endemicity were recorded following the classifications formulated at the 1950 Kampala malaria conference [3,4], initially based on spleen rates and later revised to parasite rates in children aged two to nine years old [25]. The eradication headquarters at Jinja town in Eastern Uganda housed all the survey records until 2011 when they were unfortunately destroyed.

**Figure 1:**
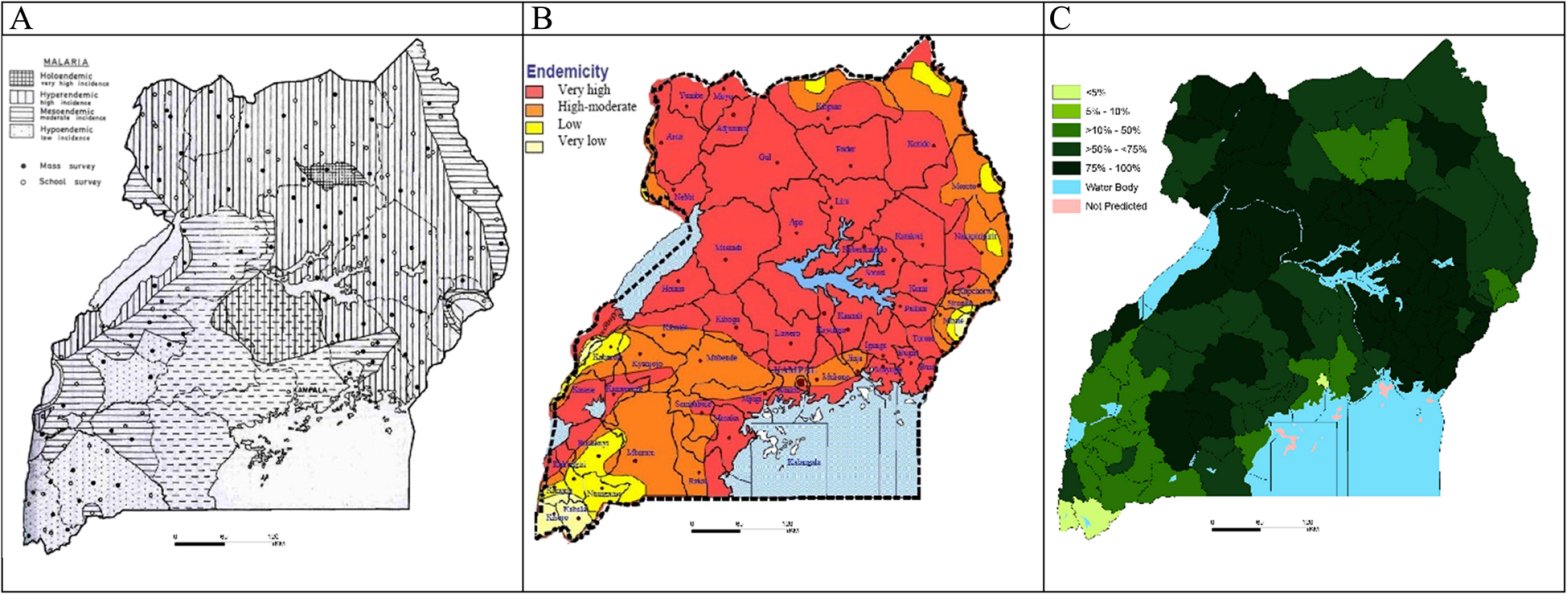
Malaria risk maps developed in A) 1960s, B) 1990s and C) 2013. **A)** Malaria endemicity regions obtained from surveys between 1965–1967 [24]. **B)** Malaria risk map used in national malaria strategies and other MoH documents from 2005. Redrawn in colour from data from1967 [24], complimented by data collected in the early 2000 [44,45]. **C)** Population adjusted mean *Pf*PR_2–10_ (PA*Pf*PR_2–10_) in 2010 [77]. To compute PA*Pf*PR_2–10_ a Bayesian hierarchical space-time model was implemented through Stochastic Partial Differential Equations using Integrated Nested Laplace Approximations for inference [13]. The covariates were used together with the parasite rate data to predict malaria risk were temperature suitability index, precipitation, enhanced vegetation index and urbanization. Continuous maps of *Pf*PR_2–10_ maps were then predicted at 1 × 1 km. The continuous maps of *Pf*PR_2–10_ and matching population density grids projected for 2010 were then used to compute the number persons likely to be positive and the populations at risk by district at each 1 × 1 km *Pf*PR_2–10_ grid location. These were then used to compute PA*Pf*PR_2–10_.

The assembled data provided an information platform necessary to produce cartographies of risk, which was used for many years after the surveys were completed (Figure 1A) [24]. MacRae’s chapter on malaria in the book *Atlas of Disease in Uganda*, edited by Hall and Langlands, first published in 1968 and revised in 1975 is an important insight for today’s malaria control/elimination epidemiologists [24,26]. Over 50 years ago, the cartography of disease distributions was regarded as a necessary public health tool. The book drew attention to the diversity of national risks, illustrated the complexity of the geographical patterns of diseases and noted that the precise limits of the distribution of many diseases were set by environmental factors [24,26]. The Atlas highlighted the interaction between altitude, open water and swamps, temperature, rainfall, vegetation, human geography, migration/immigrants, urbanization, food crops, economic factors, and population density as drivers of disease distribution. This geography of disease led to important observations, including the correspondence between Epstein Bar virus, malaria and Burkitt’s lymphoma [27-30] and the drivers of childhood diseases and malnutrition [31]. Few attempts have since been made to describe the sub-national patterns of disease through multidisciplinary studies conducted in collaboration with epidemiologists and geographers.

The intention of the pre-eradication national surveys and disease atlases was that the assembled and mapped data would serve as a baseline for future planning and monitoring. Following independence in 1962, Uganda entered a period of sustained political and social turmoil that lasted for decades. It is therefore not surprising that such national surveillance was not mounted at national or sentinel level for another 50 years.

### Re-establishing epidemiological enquiry 1995–2010

Between 1990 and 1996, a few isolated studies of the epidemiology of malaria were supported by the German development cooperation in two western Uganda districts (Kabarole and Bundibugyo). These studies were important in providing contemporary data on the micro-epidemiology of malaria in Uganda, assessing the knowledge, attitudes and practices towards malaria, its treatment and prevention as well as providing crude estimates of malaria specific mortality rates in different endemicity zones in these two districts [32]. In 1995, the Uganda Ministry of Health established a malaria control unit (MCU) [33]. However, the precise burden of the disease remained elusive during this period.

In 1998, a member of Uganda’s legislature (Parliament) requested that the Minister for Health provide the mortality estimates for malaria in Uganda disaggregated by age and sex for each of the then 39 Ugandan districts. When the Minister for Health asked the MCU team to provide the information to help him respond to Parliament’s request, the first response to the Minister was that it was not possible to provide such estimates with the available data. The health minister noted as follows: “*Why does everyone report that malaria is the leading cause of morbidity and mortality in Uganda, yet there is no evidence to support that claim”* (Kadama Patrick, persl comm.)*.* The then MCU team, like their counterparts in the first half of the Century, continued to struggle to articulate a reliable estimate of the disease burden nationwide based on routinely available health statistics, and therefore developed a ‘modelled estimate’ of 70,000 malaria deaths each year (95% confidence interval: 50,000-100,000) [33]. The Minister for Health while reporting back to the legislature elected to use the upper limit of 100,000 deaths annually, which was headline news in one of the national newspapers the following day: *“Malaria kills 100,000 Ugandans every year”* [34]. This estimate has been quoted as the country’s malaria mortality burden up to this day.

This early political plea for better data did, however, focus the attention of the national malaria control programme (NMCP) on assembling the evidence and marked the beginning of the re-establishment of the central malaria database and annual district morbidity data summaries (later reviewed in [35,36]). The need for better data was most urgent for the detection of malaria epidemics in the highland areas of Uganda, where a lack of an efficient surveillance system meant that outbreaks were detected late, had a poor response or were missed entirely. The first epidemic waves began in July 1994 in the Kabale and Kisizi regions. In July 1994, 1,684 patients were admitted to the hospital of Kisizi with a confirmed diagnosis of malaria, compared to 200 in July 1993, and 225 in 1995 [37]. Later, the El Niño Southern Oscillation (ENSO) unstable climate conditions in the Pacific during 1997–1998 led to exceptional rainfall patterns across East Africa [38] resulting in several dramatic malaria epidemics. At Kabale, the ENSO effect resulted in a malaria epidemic that lasted from February to April 1998 [9,10]. Between 1990 and 2000 regular malaria epidemics occurred in Kisoro, Rukungiri and Kabale districts in the south-western part of the country [35].

The quality, completeness and timeliness of malaria-related data from the health management information system (HMIS) slowly increased and improved over the period 2005–2010. In addition, the NMCP developed a database of all available information and survey results including those from the commercial sector. In 2008, the MCU developed the first ever M&E plan [39] and the first Malaria Indicator Survey (MIS) was conducted in 2009 [40]. However, data on inpatient malaria admissions and deaths, although systematically collected in the HMIS have rarely been analysed. This was partially addressed through the establishment of five to six sentinel hospitals with reasonably reliable data on paediatric admissions since 2000, and used in a more informed way to examine disease trends [41].

### The Uganda Malaria Surveillance Programme (UMSP)

The first attempt in over 50 years to resurrect the notion of sentinel surveillance was launched in 1997 with the establishment of the East African Network for Monitoring Anti-malarial Treatment [42,43]. However, the focus of this surveillance was not on detailed epidemiological profiling but solely on the patterns of anti-malarial drug efficacy among clinical patients at eight sentinel sites of varying malaria transmission intensity [42,43]. In the early 2000s, more basic transmission data (parasite prevalence and annual entomological inoculation rates (AEIRs) were established at these sentinel sites [44,45]. These spatially and temporally limited data were used to adapt the risk map developed in the 1960s (Figure 1A) to form an expert-opinion map of risk in 2000 (Figure 1B) and used in subsequent national strategic plans [46,47], applications for donor support [48-50] and national programme reviews [51] up to 2012.

In 2001, the Uganda Malaria Surveillance Programme (UMSP) was founded, initially as collaboration between researchers at the Makerere University College of Health Sciences, the Makerere University School of Public Health, the Ministry of Health and the University of California San Francisco. The first focus of UMSP for the period 2001–2006 was drug resistance surveillance; however the scope of the research, surveillance and the collaborators has expanded. In 2006–07, the UMSP activities were expanded to include health facility surveillance of malaria, starting with outpatient surveillance in 2006–07 and later adding inpatient surveillance in 2010. Recently, the UMSP sentinel sites have been expanded from the original six sites to twenty six malaria reference centres. In 2008, the Infectious Disease Research Collaboration in Uganda (IDRC) was created from the UMSP [52] and in 2010 the Program for Resistance, Immunology, Surveillance and Modelling (PRISM) of malaria was set up. The PRISM project builds on the platform of surveillance established by UMSP. The PRISM projects include: 1) surveillance; 2) parasite and insecticide resistance; and, 3) immunology. The surveillance project includes comprehensive surveillance at three sites (Tororo, Jinja and Kanungu), which includes cohort studies, cross-sectional community and school surveys, health centre surveillance and entomology surveys. The overall strategy is to apply a comprehensive and iterative approach to malaria surveillance so as to generate an evidence-base to help maximize the impact of control interventions across a wide range of epidemiological settings [53-57].

### Generating evidence to evaluate the impact of vector control 2000–2010

The initial focus for vector control, at the launch of RBM in Uganda, was on the distribution of insecticide-treated nets (ITNs), which started in earnest in 2002, but had failed to achieve any substantial coverage by 2010 [40,58-61]. It is notable that despite ITNs being a substantial component of all of Uganda’s national malaria strategic plans from 2000–2015 [46,47], there were limited nationwide attempts to establish any effective M&E systems to assess the impact of ITN on transmission or disease outcomes at any site in Uganda, except for the first MIS conducted in 2009 [40].

Urban malaria control, initially a key strategic intervention in the colonial period, received a renewed interest during the early 2000s. A community-based environmental management programme for malaria control was started in 2002 within two Ugandan cities (Kampala and Jinja) [62]. A detailed assessment of vector breeding sites was undertaken at two sites in Kampala (Kitebi and Kikulu) and two sites in Jinja (Police Barracks and Loco Estate). In Kampala, the interventions included filling puddles, introducing larvivorous fish and improving drainage. In Jinja, the plans focused on building and repairing drainage channels and soak-pits. Larvicides were not used. Collections of adult mosquitoes from sentinel houses suggested that there was a reduction in malaria transmission, as indicated by a drop in the number of adult mosquitoes collected. The project also set up a system of measuring malaria prevalence in the target areas and noted a reduction in malaria prevalence of 11% in the Police Barracks and 36% in Kitebi, providing evidence of the potential benefits of environmental management for reducing malaria transmission in these urban settings [62]. However, urban malaria control has not featured prominently in any subsequent national malaria strategic plans and Global Fund applications [46,47,63,64].

Since the studies at Kigezi during the 1960s, IRS had not been widely utilized in Uganda, with some exceptions of the use of lambda cyhalothrin (ICON™ 10% WP) during the epidemics in the late 1990s and early 2000s [9,10]. In 2006, the US government’s President’s Malaria Initiative (PMI) funded activities that focussed on IRS pilot projects in the south-western highland district of Kabale. Under a cooperative agreement, Research Triangle Institute (RTI) was contracted by PMI to undertake environmental and entomological surveys, procure insecticides and equipment, coordinate community education, and implement IRS activities across the whole district. IRS with lambda-cyhalothrin was carried out between June and August 2006 and post-IRS surveys were conducted in September 2006. The only data provided on the impact of this pilot project included a description of adverse events and the pre- *versus* post-malaria hospital admission rates at Kabale district hospital [65]. The following year, 45,000 households were sprayed in neighbouring Kanungu district in south-western Uganda.

The amount of detailed epidemiological enquiry on the added value of IRS to ITN remains very limited. Pre- *versus* post-spray, health facility-based, slide positivity rates were conducted at one health centre [66], rates of infection and anaemia were measured in two spray districts compared to a single unsprayed control district [67] and routine health facility data between March 2007 and October 2011 were analysed for one northern Uganda district [68]. At the time of writing this manuscript, IRS was being implemented in ten northern Uganda districts with the support of PMI. A review of the IRS programme was conducted in 2014 and observed that there was some evidence of a decline in malaria test positivity rates at health facilities in the districts where IRS was implemented compared to neighbouring districts where spraying was not done. A before and after intervention analysis showed that malaria test positivity rates dropped from 45 to 25% in the IRS districts and there was no corresponding drop in the non-IRS districts [69]. There are some interpretation issues with test positivity rates due to changing coverage but more importantly despite over six years of IRS, there are no rigorous data on the impact on vector populations, biting rates or infection risks among the infant and wider communities in these areas, in marked contrast to the ‘pilot’ investigations of IRS during the 1960s. The malaria programme’s mid-term review in 2014 admits “*there are inadequate data to support IRS planning and decision-making at all levels”* [69].

Another example of the inadequacy of epidemiological intelligence to guide strategic decisions is provided by the programme initiated by the non-governmental organization (NGO) Pilgrim Africa [70] using IRS (lambda-cyalothrin) combined with mass screening and treatment (MSAT) in two districts, Katakwi and Kumi in Eastern Uganda. MSAT was conducted using rapid diagnostic tests (RDTs) and treatment initially with a donation of artemisinin-napthaquone (Arco®), a drug not on the WHO recommended list and thus later replaced with dihydroartemisinin–piperaquine (DUOCOTEXIN®). For six to eight months, the malaria cases recorded in the routine HMIS in Katakwi district are reported to have dropped by 92% in children under five years old to 4.9% of all outpatient attendances [71]. On the basis of this alone, a draft proposal called the *Accelerated National Scale-Up of Malaria Control in Uganda (2012 – 2016)* to sustain this pilot project was presented to the Ministry of Health with a cost projection of US$2.09 billion [71], despite absence of robust epidemiological data on the short- and long-term impact of such a project.

### National population based surveys

As part of the RBM M&E initiative, national household surveys were resurrected as a means to monitor country-level malaria progress [72]. These surveys were initially embedded in the Demographic and Health Surveys (DHS) as a malaria module and were largely focussed on intervention coverage measures. In 2005, it was agreed to include malaria infection prevalence into the survey protocols. The first national parasite prevalence survey since 1967 was undertaken as part of the Ugandan DHS in 2006 [58] and subsequently repeated in 2011–12 [73]. In addition, the first MIS was conducted in 2009 [40] and another was planned before the end of 2014. Unlike previous national investigations of the epidemiology of malaria transmission, these national household surveys focussed only on children below the age of five years and sampled small numbers of children per village cluster. In contrast, the surveys undertaken in 1964–1967 examined over 120,000 people surveying all age groups in ten to 15 locations per region [13-16,20-23].

In addition to the national household survey data, there have been multiple school-based surveys undertaken in different parts of the country since 2001 [74-76] and other community-based, district-wide surveys undertaken by NGO partners and research groups. However, these data had not been systematically assembled nationally and used to define a more elaborate description of malaria transmission sub-nationally. Despite the availability of new data, the Malaria Programme Review conducted by WHO and national partners in 2010, states that “*The programme has not adopted a system for routine and periodic monitoring of malaria risk in the country*” and that one key issue was “*The lack of risk mapping (including using routine data) makes it difficult to identify populations at highest risk and targeting of interventions to these populations*” [51].

In 2013, in partnership with the NMCP, all available epidemiological data were assembled to re-map malaria risk in Uganda at a resolution of 112 districts to support the future analysis of change since 2000 and improve the understanding of previous intervention impact and future intervention targeting (Figure C [77]. This work demonstrated some changes in parasite prevalence in some districts between 2000 and 2010, but more importantly highlighted the intractability of transmission intensity despite current levels of intervention coverage. How this new epidemiological intelligence will be used for future resource planning remains to be seen. However, it is reassuring to note that the new malaria risk map has already been used in the formulation of the Uganda malaria reduction strategic plan, 2014–2020 [63] and in the funding request to the Global Fund under the new funding model (NFM) [64].

## Discussion

During the GMEP era, from the mid-1950s to the late 1960s, the use of survey data, malaria risk maps and epidemiological intelligence was a routine feature of malaria control planning in Uganda. Reconnaissance and detailed research formed the basis of decision- making. The need to assess the malaria situation and investigate the epidemiological conditions prevailing nationwide was critical for malaria programme planning. Extensive malariometric surveys were undertaken to provide a profile of risk, epidemiology and seasonality so as to guide the design of national malaria control/elimination. Impressively, such surveys were coordinated by the malaria eradication centre and technically supported by epidemiologists in the WHO country office. While this old model of doing business may be challenging in the present RBM era, because of limitations in the staffing composition and skills mix at the NMCP and the WHO country offices, there has been two decades of growth in research trained staff at universities and national institutes that can support the malaria programme.

The art and skills necessary to design malaria control based on an understanding of the spatial epidemiology were lost post-1969 and recent attempts to build a detailed understanding of risk, disease outcome and intervention impact have been less rigorous compared to the work carried out by the malariologists of the 1950s and 1960s. The current designs of MIS or DHS, although useful in providing valuable information on intervention coverage, are inadequately designed to provide reliable spatial data on species-specific and age-specific infection prevalence and do not include an entomological component.

During the RBM era the detailed evaluation of vector control impact has been absent in Uganda, despite more recent investment in sentinel surveillance. The added value of IRS combined with ITN remains unclear in different transmission settings or when universal access to LLINs has been achieved [78-80]; the impact of ITNs on transmission and disease rates at current levels of coverage and transmission intensity remain unknown, the role of MDA, while investigated in detail during the 1950s has been poorly defined during recent interventions. There is presently a renewed interest among development partners and malaria researchers in the role of MDA in areas of intense transmission. Any future pilot experiments of vector control and parasite prevention in Uganda should be accompanied by robust epidemiological, entomological and community acceptability evidence, at least to the level of detail provided in the 1950s and 1960s. Moreover, pilot studies should also assess the long term rather than short-term impact of MDA [81]. This evidence generation, synthesis and policy implication cycle needs further development in Uganda.

The diversity of malaria transmission in Uganda has not been used to inform a tailored national strategy. Most notable are the conditions that prevail in urban centres, home to one in five of Ugandans, and where further trials of innovative integrated vector management in densely populated areas are urgently needed.

One area of malaria intelligence that has to a large extent been used effectively to inform national policy are data generated on parasite resistance to first and second-line anti-malarial medicines [82-85]. However, these sentinel data need increased investment to improve six- to eight-hourly parasite density sampling or molecular surveillance to detect emerging artemesinin resistance [86]. Further intelligence data that has been effectively used to inform the IRS policy are data on vector resistance to public health insecticides [87,88]. However, national patterns of vector species ecology and the changing vector bionomics in the face of widespread ITN use remains poorly defined. Vector distribution, their peak biting times and predominance of indoor *versus* outdoor biting is critical in assessing what new paradigms are needed for malaria vector control in Uganda.

The precise malaria mortality burden in Uganda has remained unknown since the 1920s, all past and recent estimates are based on a modelled prediction of sparse national data: 100,000 annual deaths in 1998 [32,45,46], modelled data from outside of Uganda applied to Uganda’s population, 23,126 annual deaths [89] or 17,000 annual deaths [90] or data reported by the national health information systems, 10,500 annual deaths [63]. Improving the use of routine statistics is paramount; however, interpreting incomplete data of ‘malaria’ among semi-immune populations continues to be a challenge [91,92]. Infection prevalence is a less ambiguous metric [13] and there is a renaissance in rapid, school-based sampling of malaria infection to monitor change at sub-national levels [93,94].

There is a tendency among donors and national malaria control programmes to make clear demarcations between research and M&E, leading to limited funding for some of the critical areas needed for robust intelligence. Ideally these two areas should be intimately connected. Basic requirements for national malaria control planning are: a) the epidemiological basis of parasite-vector-host transmission, and b) what interventions work across the diverse national malaria ecology. Neither has been properly defined during the rapid expansion of funding for malaria control in Uganda during the RBM era. Both were extensively conducted during the GMEP era at a time when there was limited funding to implement tailored and tested control. As funding becomes harder to access post-2015, a more rational basis for malaria funding is required. More data rather than less will be necessary to improve value for money. The Global Fund’s allocation for M&E under the round-based system was ring fenced at approximately 10% of the entire grant. The Global Fund’s NFM is commendable because it attempts to align funding windows with national planning cycles and furnishes upfront indicative funding that is available to a given country for the three diseases (HIV/AIDS, tuberculosis and malaria). However, the NFM has removed the ring fencing that previously protected funding for M&E. Whether this removal of ring fencing will reduce the prioritization of M&E is yet to be seen. However, all stakeholders should ensure that the funding for surveillance, M&E should increase rather than decrease. Over the next five to ten years, accountability, impact analysis, financial business cases supported by a culture of data use should become the new paradigm by which malaria programmes, governments and their development partners operate.
